# SARS-CoV-2 vaccine boosters: The time to act is now

**DOI:** 10.1371/journal.pmed.1003882

**Published:** 2021-12-09

**Authors:** Maya Petersen, Joshua Schwab, Diane V. Havlir

**Affiliations:** 1 University of California, Berkeley, Berkeley, California, United States; 2 University of California, San Francisco, San Francisco, California, United States

## Abstract

We have a new and unprecedented opportunity to mitigate the suffering, death and inequities of COVID-19 in the United States with vaccine boosters—if we deploy them effectively, rapidly, and widely with simplified messaging to all eligible adults.

## Abstract 2

Maya Petersen and co-authors discuss priorities for vaccination against SARS-CoV-2 in the United States and beyond.

SARS-CoV-2 vaccines currently approved in the US are highly effective and provide a powerful tool for pandemic control. However, vaccine-induced protection from SARS-CoV-2 infection wanes substantially over time, including among persons aged under 65 years [1]. Strong evidence also supports the effectiveness of vaccine boosters for adults of all ages. Observational data from Israel, where widespread boosting of persons vaccinated with Comirnaty (BNT162b2) began in July 2021, show profound benefits of boosters for prevention of infection, hospitalization and death [2]. The benefits of boosters seen in these and other observational data were recently confirmed in a large, multisite randomized clinical trial of a BNT162b2 booster [3].

The Centers for Disease Control (CDC) extension of booster eligibility beyond adults with higher medical or environmental risks to all adults is supported by individual-level medical tradeoffs [4]. Even among males and females 18–29 years of age, COVID-19 hospitalizations prevented by boosters would outnumber cases of booster-associated myocarditis under a range of assumptions [5]. In terms of longer-term risks and benefits, some have raised concern regarding unknown effects of inflammation due to repeated vaccine injections. There is no evidence to support this claim, but there is evidence that immune effects of the virus itself, even in the setting of mild infection, can lead to longer-term debilitating consequences. In short, there are good arguments for ensuring that healthy young people have access to boosters to protect their own health.

Following the emergence of the Omicron variant, the CDC updated their guidelines from allowing to recommending boosters for all adults [4]. There are theoretical reasons that detection of the Omicron variant strengthens the argument for universal boosting, yet very little remains known about this variant and its epidemiologic characteristics. However, and crucially, even in the absence of the Omicron variant, two data-driven arguments, in addition to those based on individual medical risk and benefit, strongly support widespread boosting for young and middle-aged healthy adults.

First, there are profound population-level benefits of boosting for epidemic control. Vaccinated people can be infected, and can infect other people, including others who are vaccinated [6]. Waning immunity and rising contact rates mean that many parts of the US are at risk of major winter surges in both cases and hospitalizations. Widespread boosting now has the potential to mitigate these surges. San Francisco provides a case study. At time of writing, 76% of city residents were fully vaccinated and COVID-19 mortality remained among the lowest in the country [7]. However, San Francisco is not “out of the woods” for this pandemic. Our model, publicly available and used to guide the COVID-19 response locally and in other regions since March 2020, shows the potentially dramatic impact of widespread vaccine boosters over the coming months (Fig 1) [8]. Based on model projections prior to the emergence of the Omicron variant, if booster deployment outpaces waning immunity from infection, a resurgence in hospitalizations can be prevented even as risk behaviors increase substantially. However, if waning outpaces boosting then a resurgence in hospitalizations is likely, even with less behavior change as we head into the holidays. Importantly, this is true even if no or minimal further waning occurs in vaccine effectiveness against severe disease. The importance of widespread boosting to prevent a resurgence in hospitalizations will vary by region, and depends on the initial vaccine rollout and the timing and extent of prior infections. Results from our and others’ models support the crucial role of widespread boosting for epidemic control in much of California and in the UK [9].

**Fig 1:**
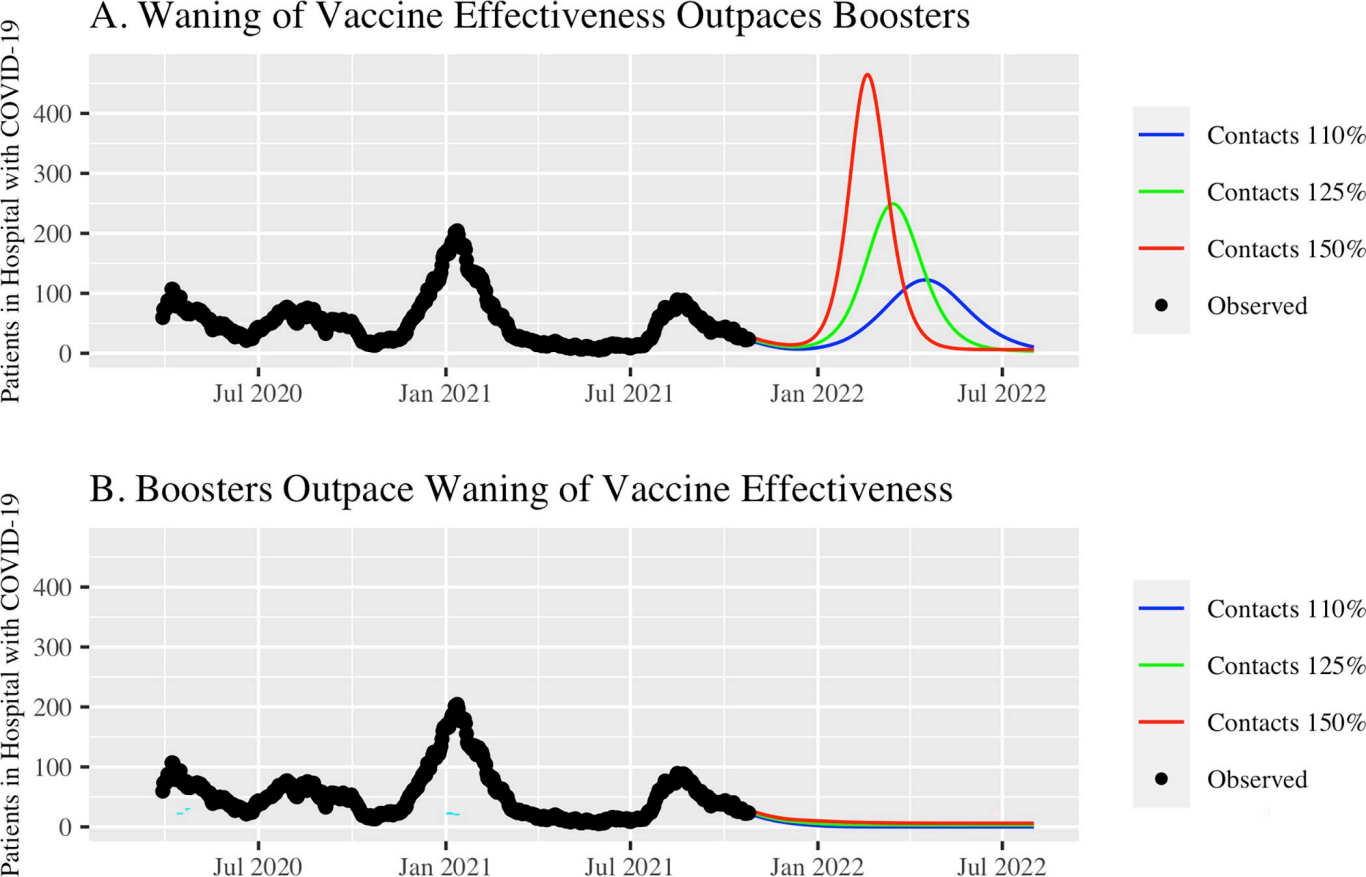
Observed COVID-19 hospital census in San Francisco through October 25, 2021 (black dots) and projected hospital census over coming months (colored lines) assuming differing levels of risk behavior change (effective contact rate increases to 110%, 125% and 150% of level on October 25, 2021) if A) waning of vaccine effectiveness outpaces booster roll-out; B) booster roll-out outpaces waning of vaccine effectiveness. Projections based on the Local Epidemic Modeling for Management and Action (LEMMA) model, calibrated to publicly available data and reflecting the epidemic pre-Omicron. Detailed description, source code, and outputs available at https://localepi.github.io/LEMMA/.

Second, effective and widespread rollout of boosters now is crucial to avoid further exacerbating the disproportionate impacts of the COVID-19 pandemic on the lives and livelihoods of communities of color. Black, Hispanic, and American Indian/Alaskan Native persons have experienced at least double the risk for hospitalization and death of other races/ethnicities, with impacts that ramify through families and communities [10]. Further, even mild infections prevent front-line workers from doing their jobs and can create a cascade of financial, social and health consequences. The structural inequities that drive disparities in both transmission and severe disease are still with us, suggesting that communities of color will once again bear a disproportionate burden of new surges in SARS-CoV-2 transmission, particularly given the ample time for waning of immunity that has now elapsed since early waves of infection in many of these communities.

In many parts of the country, vaccine coverage remains lower in communities of color. Person-for-person, the benefits of initial vaccination outweigh those of boosting, and continued efforts to increase initial vaccination coverage must continue. However, the drive to get all persons vaccinated is complementary to, not at odds with, efforts to achieve widespread booster coverage. Simplified booster eligibility combined with low-barrier approaches grounded in community partnerships provide a means to deliver both initial doses and boosters, and to avoid creating a false dichotomy between the two. Further, by enabling continued epidemic control as contact rates increase, widespread boosting is itself a tool to protect persons who remain unvaccinated.

The argument for widespread boosting in the US exists in parallel with the immediate ethical imperative to improve global vaccine access. The lack of global access to vaccines is appalling and indefensible. In the majority of countries in Africa, less than 10% of the population have been fully vaccinated [11]. Vaccine supply has dramatically accelerated; distribution is lagging. We advocate for a massive ramp-up of global vaccine distribution (including boosters) and an immediate push for boosters in the US.

How should the booster roll-out be implemented? Unlike the first quarter of 2021, vaccines are not in short supply. The CDC recommendation to allow either the same vaccine for boosting (matching) or an alternate vaccine (mixing) is supported by immunologic studies of adults [4]. This boosting approach simplifies implementation. Therefore, recommendations for non-immunocompromised persons should be simple: all persons fully vaccinated should receive a booster shot after 6 months. Messaging should also be simple: We have excellent vaccines, they need to be given more than once, and they may have to be given on a regular basis in the foreseeable future.

Over the holidays, many people will want to gather with friends and family. This year we have both highly effective vaccines, including for children 5 years of age and older, and highly effective vaccine boosters. With simple eligibility and clear science-driven public health messaging, we can use these measures to help control transmission of SARS-CoV-2, maintain open economies and cultures, and work to mitigate the profound health inequities exacerbated by COVID-19. The time to act is now.
